# School principals’ proposals for preventive actions to improve mental health

**DOI:** 10.1186/s13690-024-01481-4

**Published:** 2025-01-23

**Authors:** Roger Persson, Ulf Leo, Inger Arvidsson, Kerstin Nilsson, Kai Österberg, Carita Håkansson

**Affiliations:** 1 Department of Psychology, Lund University, Lund, SE-22100 Sweden; 2 Centre for Principal Development, Umeå University, Umeå, Sweden; 3 Division of Occupational and Environmental Medicine, Lund University, Lund, Sweden; 4 Department of Public Health, Kristianstad University, Kristianstad, Sweden

**Keywords:** Assistant school principal, Exhaustion, Stress, Stress management, Work-related stress

## Abstract

**Background:**

School principals face a demanding work situation that puts them at risk for stress-related poor mental health. Ideally, preventive actions should be based on knowledge about the underlying notions that motivate action. However, knowledge about prevention areas and suitable initiatives for school principals is scarce or lacking, leaving key stakeholders without the overview necessary for effectively engaging in preventive actions.

**Objective:**

To describe school principals’ proposals for suitable target areas and preventive actions that may decrease their workload and improve their mental health.

**Methods:**

An aggregated analysis was conducted using data from a larger project involving two national surveys (*N* = 2871), nine group interviews (*N* = 39), and eleven workshops (*N* = 270). The project was designed a priori to examine multiple questions, including identifying target areas and collecting proposals for preventive actions.

**Results:**

Forty-four proposals for preventive actions spanning various levels and six major themes were identified: Role demands and role conflicts (8 proposals); Organizational support (6 proposals); Resources and resource allocation (3 proposals); Continued professional development and coaching (2 proposals); The principal as a coworker (6 proposals); Personal actions (19 proposals).

**Conclusions:**

The considerable number of proposals and their content reveal that preventive actions are needed at all levels of the educational system and that the school principals’ personal resources matter for improving their work situation and mental health. The pattern of results also underscores the importance of having a deep understanding of what problems to address, and in which context they unfold, before engaging in preventive actions.

**Table:** 

**Text box 1. Contributions to the literature**
• Contextually relevant knowledge about potential prevention areas and associated suitable initiatives for improving school principals working conditions and mental health is scarce or lacking.
• The school principals’ work situations pose many organizational challenges and mental health risks that needs to be addressed by tailored preventive actions that take local conditions and the principals’ personal resources into account.
• Stakeholders on the national, school owner, and local school unit level need to ensure that school principals’ working environment and mental health receive the attention they deserve.
• Future preventive actions need to be carefully selected and coordinated among stakeholders at various levels.

## Background

As part of an extensive educational system, school principals’ actions may influence teachers and students and may eventually affect public health. It is therefore concerning that studies from a wide range of countries [1–5] have shown that school principals face a stressful work situation that puts them at risk for work overload, chronic stress, and eventually, occupational conditions such as burnout [6] or medical and psychiatric problems such as depression, anxiety and exhaustion disorder (ED; ICD-10-SE) [7] and other forms of stress-related poor health (e.g., cardiovascular disease) [3, 8]. Because overburdened school principals are less likely to fulfil their managerial role, they might not live up to their duty to be responsible for the internal organization, pedagogical development, and teachers’ and students’ work environment [9]. To achieve their mission and be able to work until retirement, school principals, like others, need proper work-related organizational preconditions, motivation, and good health [10, 11].

According to the Organisation for Economic Co-operation and Development (OECD) mandated Teaching and Learning International Survey (TALIS) [12, 13], stress is a major factor influencing Swedish school principals’ job and career satisfaction, and their main stressors are having too much administrative work, having extra duties due to absent school staff, and having to accommodate students with special needs. In addition, roughly 25% of Swedish school principals express a symptomatology including signs of exhaustion that, if sustained, might lead to poor health [14]. They also rate their work ability slightly lower than a stratified random national sample [15], and both governmental agencies [16] and researchers [17, 18] have reported that turnover among Swedish school principals is too high and disrupts organizational continuity.

While the situation would seem to warrant preventive action, it is not evident what types of action are needed or desired. Ideally, however, preventive actions should be based on knowledge about the underlying notions that motivate action and a proper understanding of the context. However, knowledge about relevant prevention areas and associated suitable initiatives is scarce or lacking, leaving occupational health services, employers, and authorities without the overview necessary for effectively engaging in preventive actions.

Apart from the difficulties associated with not knowing where or how to intervene, a complicating circumstance is that school principals’ work consists of managing relations and the expectations of various stakeholders on multiple levels (e.g., the National Agency for Education, the Schools Inspectorate, the Swedish Working Environment Authority, media, politicians, parents, teachers, and students) [19]. Thus, psychological and social processes may cause a wide range of daily difficulties and ad hoc stressors that justify different preventive actions meant to prevent these difficulties and stressors – alone or in concert – from giving rise to accumulated negative health effects. Adding to this complexity are variations in systemic and organizational preconditions resulting from the fact that both public and private actors (e.g., share holding companies, cooperatives, foundations) may own and operate schools [9] as well as that Sweden has a system of dual governance. While the state governs via rules and regulations, national curricula, and syllabuses, the 290 municipalities, independent school owners, and local school units are the implementing bodies [9].

To better understand the possibilities for improving Swedish school principals’ work environment and mental health, a nationwide research project was carried out from 2018 to 2021. The project was designed a priori to examine multiple questions, including identifying target areas and proposals for preventive actions. In the present paper, we describe the results from an aggregated analysis of the data obtained within this project. The aim is to describe the school principals’ proposals for suitable target areas and actions for preventive efforts that may decrease their workload and improve their mental health.

## Methods

### Study design

An aggregated analysis of findings from six research studies [14, 15, 19–22] and two reports in the Swedish language [23, 24] was performed. All data were collected within a larger project that involved two national surveys (in 2018 and 2019), nine group interviews (in 2019), and eleven workshops (in 2020). The Regional Ethical Review Board in Lund, Sweden, and the Swedish Ethical Review Authority approved the study protocols (reg. no. 2018/247 and 2023-01497-02, respectively). In accordance with the study protocols, the participants gave their informed consent when they began participating in the studies.

### Participants

The survey participants (*N* = 2781) came from all 21 counties and 277 of Sweden’s 290 municipalities; they had completed the web survey at least once in 2018 (*n* = 2317) or 2019 (*n* = 1992). Given the lack of an accessible official census on school principals, the participants were recruited via a compiled e-mail list with addresses containing school principals who, during the period 2008 to 2017, had participated in training programmes funded and arranged by the Swedish National Agency for Education. Demographic data for participants in the two web surveys are presented in Table 1. Of the 9900 invited, 4640 potential responders either actively declined (*n* = 2007) or accepted (*n* = 2633) participation (i.e., a 47% response rate), and 2317 individuals completed the survey (i.e., a 23% response rate in relation to all invited and 50% in relation to those who actively responded to the e-mail invitation). In 2019, all but one retiree among the responders from 2018 were reinvited, and 1528 completed the second survey (i.e., 66% of the responders in 2018). In addition, 5149 individuals who did not respond at all to the first invitation were reinvited. Of these, 1506 (i.e., 29%) broke silence and made an active decision to decline (*n* = 882) or accept (*n* = 624) participation, and in the end 464 completed the 2019 survey. Of those who completed the survey in 2018, 1797 (78%) provided at least one free-text answer and 1177 (51%) answered all three open-ended questions in the 2018 survey. The response rate for the individual survey questions a, b, and c were 65% (*n* = 1518), 71% (*n* = 1655), and 60% (*n* = 1383), respectively. See “Data collection and analysis” for the wording of the questions.

**Table 1:** Descriptive participant data across the years 2018 and 2019

	Year					
	2018				2019	
	Total (*N* = 2317)		Responders to questions on measures (*n* = 1797)		Total (*N* = 1992)	
**Age** (Years; Mean, SD)	49	7	50	7	50	7
**Gender**	**N**	**%**	**N**	**%**	**N**	**%**
Female	1803	78	1400	78	1564	79
Male	510	22	395	22	425	21
Other/do not wish to disclose	4	0	2	0	3	0
^**1**^ **Job title**						
Principal	1786	77	1419	79	1587	80
Assistant principal	408	18	293	16	331	16
Other title	123	5	85	5	74	4
**School level**						
Pre-school	631	27	482	27	533	27
Pre-school and compulsory school	116	5	100	6	95	5
Compulsory school	1000	43	785	44	901	45
Upper secondary school	347	15	277	15	299	15
Adult education	163	7	121	7	118	6
Other	60	2	32	2	46	2
**Years as a school principal**						
0 to 3 years	445	19	341	19	160	8
> 3 years up to 5 years	519	22	410	23	461	23
> 5 years up to 10 years	797	34	604	34	791	40
More than 10 years	556	24	442	25	580	29
**School owner**						
Municipality	1787	77	1410	79	1529	77
Other	530	23	387	21	463	23

SD = standard deviation

^1^ Includes the job titles pre-school director (*n* = 569) and principal and pre-school director (*n* = 70), which from July 1, 2019, were renamed “principal”

A total of 2781 unique individuals responded to at least one of the two surveys, and 1528 individuals participated both years

The participants in the nine group interviews were selected from the survey participants. To ensure inclusion of perspectives from northern, central, and southern Sweden, school principals were strategically selected from three municipalities in these geographical areas to represent pre-schools, compulsory schools, and upper secondary schools. Altogether, in the 2018 survey, 1348 (58%) individuals indicated their willingness to participate in group interviews. Of these, 48 school principals were invited and 39 were interviewed (i.e., an 81% response rate). The mean age was 50 years (range 35 to 65 years), about 75% were women, 94% percent worked full-time, and approximately 66% had worked overtime a few times a week during the past 12 months. On average, each principal was responsible for 31 employees.

Recruitment of participants to the eleven workshops varied. The first workshop was conducted as a voluntary activity at a national school principal conference in February 2020, and the response rate cannot be estimated. Due to Covid restrictions, the other ten workshops were conducted digitally between August and October 2020. For three workshops, we reinvited the 39 participants from the group interviews, whereas the workshop participants were recruited from the national school principal programme run by Umeå University. Of the 39 reinvited participants, 27 contributed to the workshops (i.e., a 69% response rate). In the seven subsequent workshops, 201 individuals from seven course groups in the mandatory national school principal program (*n* = 39, *n* = 28, *n* = 28, *n* = 27, *n* = 27, *n* = 26, *n* = 26) were invited to participate in school-level specific workshops and all decided to contribute (mean age 47 years; and 83% were women). Of these, 120 were school principals, 79 assistant school principals, and 2 individuals had a different title but school principal duties.

### Data collection

Three rounds of qualitative data collections were performed. The rounds were designed to augment and supplement each other. In 2018, the first round comprised open-ended responses in a survey and sought to collect the most important suggestions from all responders. In 2019, the second round comprised nine group interviews and sought to deepen suggestions raised in the survey. In 2020, informed by the survey and group interview data, the third round comprised eleven workshops and sought to identify concrete suggestions on how to improve the school principals working environments.

In the first round (i.e., 2018 survey), participants were asked to respond to three voluntary open-ended questions that were preceded by a brief explanation: “*One of the purposes of the research project is to propose ideas and measures for improving school principals’ working environment. We therefore ask for your suggestions on measures*”. The respondents were then asked to provide what they perceived as the three most important proposals in response to the following questions: (a) *Which measures on the national level* *could improve your work environment? (b) Which measures on the* *school owner level* *(your municipality/independent school/ pre-school) could improve your work environment?* (c) *Which measures on the* *school unit level* *(school*, *pre-school) could improve your work environment?* For each question, the response length was limited to 480 signs.

In the second round (i.e., nine group interviews), the semi-structured interview guide aimed to facilitate the exploration of how and when principals experience external expectations and how these expectations impact on their work situation, leadership, and mental health. In addition, the interview guide also aimed to identify the types of resources and support the principals perceived as necessary to improve their situation. All interviews were led by one of the authors (UL) who always paired up with another author (i.e., IA, CH or RP). All interviews were recorded and transcribed in verbatim.

In the third round (i.e., the eleven workshops), three overarching questions were raised and addressed: (a) *Which concrete measures can the* *state take to improve your work environment and well-being*? (b) *Which concrete measures can be taken at the* *school owner level* *to improve your work environment and well-being*? (c) *Which concrete measures can you take* *yourself* *to improve your work environment and well-being?*

The workshop at the national school principal conference was led by UL and RP whereas the seven workshops entailing participants from the national school principal programme were led by UL alone. The data from these eight workshops were collected groupwise using the Mentimeter software (Menitmeter.com) allowing for multiple responses limited to 200 signs each. In contrast, the data from the three workshops with re-invited group interview participants were recorded and transcribed. These workshops were led by UL who teamed up with another author (i.e., IA, CH or RP) .

### Data analysis

The free-text answers from the 2018 survey and the Mentimeter data from eight workshops were subjected to a thematic analysis [25]. Most of the responders to the open-ended questions in the survey provided one or two suggestions on measures on how to improve the principal’s working environment on the different levels in the education system. The responses were often formulated as short sentences. However, incomplete sentences, short phrases, or just single words were also common. The Mentimeter software similarly favoured workshop participants to respond in short sentences and phrases or just single words. In contrast, the data from the nine group interviews and the three workshops with the re-invited group interview participants were longer and more elaborate.

The group interview and workshop data were analysed using both thematic and content analysis contingent the purpose of analysis [25]. The group interview and workshop data were coded using the NVivo 12 software, whereas the analysis of the open-ended survey responses was managed via word, excel, and the IBM SPSS 29 software’s. The primary coder for both the survey, group interview and workshop data were UL who consulted with other authors when needed (i.e., IA, CH, and RP). UL has an extensive experience of working with professional development for school principals, and a background as a teacher and principal with an insight in principals’ practice.

The aggregated analysis, combined the individual results from the three qualitative data collections by stacking the proposals and interpreting them in the general context provided by the authors a priori knowledge and our previous findings in this project [14, 15, 19–22]. However, in alignment with the study design, the workshop data was assigned a greater weight in the final selection of proposals. In detail, a list of proposals were compiled by the primary coder (UL) to represent recurring proposals in the form of themes and single proposals based on their similarity across the three rounds of data collections. Proposals that were very infrequent, vague, or only addressed the physical work environment without further specification or had no bearing on the school principal’s leadership role, health and well-being were discarded given the general aim of the research project. Next, the compiled list of proposals was subjected to internal peer-review in the author group (all authors) that checked that the selection of proposals appeared correct and reasonable considering each authors own understanding and knowledge of the material. This process resulted in a further distinction in which suggestions relating to the situation were grouped and separated from suggestions relating to the person. Hence, while the different data collections methods contributed with a cross-validation of the proposals, the internal peer-review process ensured that the final themes and proposals had been compiled adequately and that they as accurately as possible accounted for the principals’ proposals.

## Results

The aggregated analysis of survey data, group interviews, and workshops identified six major themes covering 44 proposals of preventive actions for improving school principals’ work environment and mental health. Five themes contained 25 proposals targeting the situation, whereas the last theme contained 19 proposals targeting the individual school principals.

The five themes targeting the situation were:

- Role demands and role conflicts (8 proposals).
- Organizational support (6 proposals).
- Resources and resource allocation (3 proposals).
- Continued professional development and coaching (2 proposals).
- The principal as a coworker (6 proposals).

The single theme targeting the individual was grouped into three categories:

- Set limits and prioritize (10 proposals).
- Distribute leadership in different ways (5 proposals).
- Invest in foresight and professional development (4 proposals).

Tables 2, 3 and 4 summarize the preventive actions aimed at the situation, whereas Table 5 summarizes the proposed personal actions.

**Table 2:** School principals’ proposals for preventive actions: role demands and role conflicts

Proposal	Explanation
**Role demands and role conflicts**	**The school principals wished…**
1. Clarify regulation of school principals’ duties at the state level.	… to alleviate their stress by gaining clearer and more transparent governmental regulation of their duties. The Swedish Education Act was perceived as leaving too much room for local interpretations that could create role conflicts.
2. Clarify how national politics relate to school principals’ duties.	… for transparent and predictable relationships between national politics and the local school units. Detailed political proposals on the national level were sometimes perceived to clash with local matters and in the process cause stress and frustration among school principals.
3. Clarify the regulation of school principals’ duties on the school owner level.	… for inclusion and greater possibilities to influence decisions in the local chain of governance. That many school owners have centralized their organizations and introduced several layers of leaders between the principal and the owner is sometimes perceived as infringing on school principals’ feelings of autonomy.
4. Clarify the regulation of school principals’ duties on the local political level.	… for a more transparent and predictable relationship with the local political level, as they sometimes felt stressed and frustrated by detailed decisions taken by local education committees that stripped them of their own local responsibility.
5. Review local delegation orders and delegate work to other occupational groups.	… to increase their focus on their core tasks and reduce their stress and workload by delegating work to other employees, particularly duties for which they are not trained (e.g., property issues and monitoring the physical work environment).
6. Give school principals fewer and specific goals and long-term goals for the school.	… for fewer, well-defined long-term goals from local politicians and school owners; In contrast to short-term goals that were often perceived as frustrating because they interfered with continuity and long-term development.
7. Develop relationships with local educational boards or committees.	… for improved mutual understanding and improved professional relationships with local political actors, as they felt local politicians too often failed to understand their complex work tasks.
8. Change routines for handling notifications to the Schools Inspectorate.	… for clearer guidelines and routines to deal with notification cases to the Schools Inspectorate, which requires thorough documentation; cases are often perceived as burdensome and stressful as they may linger for long periods.

**Table 3:** School principals’ proposals for preventive actions: organizational support and resources and resource allocation

Proposals	Explanation
**Organizational support**	**The school principals wished…**
9. Review support functions at the school owner level.	… to decrease their workload by receiving more support and hands-on service to better deal with, for example, absent students, economic issues, and human resource issues (e.g., employments and rehabilitation issues).
10. Create venues for increased self-governed exchange between school principals.	… for more collegial support and informal self-administered venues that facilitate exchange with other manager colleagues on matters that are not covered by formal management teams.
11. Increase local management support.	… to decrease their administrative burden by establishing, or increasing, daily management support at the local school unit.
12. Regulate the information flow with guidelines.	… to receive help in handling the mentally taxing flow of digital information and to establish communication plans and guidelines for how to behave digitally.
13. Better coordination of IT systems; increase IT systems’ user friendliness.	… for better co-ordinated, compatible, and more user-friendly IT solutions, as this would reduce associated and common work environment problems.
14. Increased administrative support for handling notification cases to the Schools Inspectorate.	… for increased administrative support to decrease the workload and stress related to work with notification cases involving the Schools Inspectorate.
**Resources and resource allocation**	**The school principals wished…**
15. Increase the government’s economic control.	… that the state would assume clearer responsibility regarding the distribution and management of financial resources, as this would improve equality across school units and dispel concerns, worry and frustration.
16. Reform and simplify access to, and use of, state subsidies and accounting.	… for reformed state subsides, as the current system was perceived to be cumbersome, short-term, and not always aligned with local needs, often leading to time-consuming and frustrating administration.
17. Increase equality across school units.	… to reduce the extra workload associated with working in segregated and challenged socioeconomic areas through stronger financial management that should balance the effect of socioeconomic differences and segregation across schools.

**Table 4:** School principals’ proposals for preventive actions: continued professional development and coaching and principal as a coworker

Proposal	Explanation
**Continued professional development and coaching**	**The school principals wished…**
18. Increase access to introduction programmes and mentorship for new principals.	… for introduction programmes and organized mentoring to reduce stress caused by the transition from a teaching position to school principal.
19. Ensure permanent access to continued education on the national level.	… for continuous education and professional development beyond the extant national school principal programme, as this was perceived to facilitate their work and professional development.
**Principal as a coworker**	**The school principals wished…**
20. Ensure regular dialogues with one’s immediate supervisor and meetings focusing on school principals’ own work environment.	… for an increased focus on their own work environment, regular dialogues with their immediate supervisor, an increased understanding of each other’s work roles, and meetings about their own work situation.
21. Perform regular risk analyses of school principals’ work situation.	… for increased risk management activities, closer monitoring of workplace risks, and regular health examinations.
22. Regulate, or recommend, a maximum number of employees per principal.	… to reduce their role demands through governmental guidelines, or regulations, regarding their span of control (i.e., how many employees they are leading).
23. Strengthen opportunities to share leadership	… to increase chances of success and to combat feelings of uncertainty and being alone in their mission, by having opportunities to share leadership with colleagues on the same level.
24. Limit the number of activities a principal can oversee.	… to decrease the workload and stress by limiting the number of activities school principals may oversee, as activities may be geographically dispersed and require different types of leadership.
25. Review principals’ salaries so that there is a difference between them and teachers’ salaries.	… that school principals’ and teachers’ salaries would differ, because it was perceived as unjust, frustrating, stressful and a loss of status when teachers with fewer responsibilities due to governmental reforms got higher salaries than they did.

**Table 5:** School principals’ proposals for preventive actions aimed at themselves and other school principals

Proposal
**Set limits and prioritize**
1. Separate work and personal life to allow for necessary recovery time.
2. Think and choose when to be available and present.
3. Think about communication and remember that you cannot always be available.
4. Take command of your time - own your schedule and almanack.
5. Prioritize high-quality meetings and reduce the total number of meetings.
6. Practice operational slowness - do not produce solutions immediately when employees are asking for your advice.
7. Improve your own ability to prioritize between work tasks.
8. Make time for reflection and reflect at regular intervals.
9. Signal clearly to staff how your own time is used, what your priorities are, as well as what kinds of prioritization are part of your work role as a school principal.
10. Signal clearly to staff and colleagues and set limits when work becomes overwhelming.
**Distribute leadership in different ways**
11. Delegate specific tasks to others more frequently.
12. Distribute developmental leadership to teachers.
13. Share leadership with colleagues at the same level.
14. Establish specialists among staff in various work areas.
15. Participate in collective learning with staff and other leaders.
**Invest in foresight and professional development**
16. Engage in long-term planning (e.g., use an annual wheel as a tool).
17. Ensure long-term collegial support from other principals.
18. Ensure regular professional coaching.
19. Strengthen competence through continuous training and education.

In brief, regarding the suggestions targeting the situation, the school principals’ proposals concerning role demands and role conflicts revealed that they had multiple roles and both stimulating and demanding work tasks relating to accountability for results and quality, personnel, organizational development, and the work environment. However, role conflicts arose when different demands were pitted against each other. Reports of struggles between administrative work and managing pedagogical development for staff and students or perceiving a lack of time for communicating with employees and performing forward-looking strategic work were common. The proposals concerning support in the organization showed great variation in support that was seemingly contingent on the size of the organization, in that there were advantages and disadvantages associated with both small and large organizations. Furthermore, the quality of support and how it was communicated were described as decisive features determining whether specific activities are perceived as support or simply as a nuisance. The proposals concerning resources and their allocation primarily revealed perceived shortcomings. Examples are lack of financial resources, understaffing, difficulties in hiring qualified teachers, or inadequate school premises. The proposals concerning continued professional development and coaching mainly favoured initiatives that could provide long-term support. The proposals concerning the principal as coworker showed that, as employees, school principals were sometimes forgotten and that their work environment did not receive much attention from their superiors.

The proposals concerning the school principal as an individual revealed a desire to reduce the workload to create manageable stress levels and to enable long-term and proactive educational leadership by, for example, engaging in self-reflection, developing the individual’s ability to prioritize, delegate work tasks, share leadership, make long-term plans and engage in professional development (Table 5).

## Discussion

The participating school principals made many thoughtful proposals for preventive actions they believed could improve their work situation and mental health. The diversity and content of their proposals suggest that they experience complex patterns of work-related psychosocial exposures that are likely to resist easy translation into a few broad, uniform preventive actions. The abundance and content of proposals also underscores the difficulties of identifying the most relevant areas for preventive actions and prioritizing among them. Moreover, as observed during the nine group interviews and eleven workshops, individual proposals are not necessarily required, or suitable, everywhere. All municipalities, school owners, and school units have different preconditions and often face different challenges.

Jointly, on an overarching level, the proposals seem to express a desire for primary and secondary preventive actions targeting both external circumstances and individual factors that promote proactive and sustainable educational leadership (i.e., healthful leadership with a long-term perspective) [26]. Because the school principals had previously been encouraged to search for proposals for preventive actions on the national, school owner, and school unit level in our line of questioning in the 2018 survey, group interviews and workshops, it is not surprising to find proposals related to these levels. However, preventive actions at adjacent, or intermittent, levels were also proposed (e.g., the political level). Interestingly, proposals regarding how to manage potentially health hazardous forms of social exchanges and maltreatment that are known occur in schools (e.g., harassment, bullying, threats about violence, or violence) were very few and not uniform regarding their content. The absence of such proposals should, however, not be interpreted as these phenomena are unimportant or not present in the Swedish school environment. It is merely to say that these issues seldom made it to the school principals top three list of proposals in the survey and that they did not stand out in the group interviews or workshops.

The fact that the proposals for preventive action were frequently related to perceived role conflicts and role demands aligns well with earlier findings. We have previously reported associations between role conflicts and decreased work ability [15], increased turnover intentions [20], and exhaustion symptoms [21]. Moreover, the proposals for preventive actions concerning increased support align well with our previous findings in related subsamples of school principals. For example, the experience of having supportive managerial colleagues seems to be beneficial to both work ability and mental health [15, 21]. The importance of also having regular dialogues with one’s immediate supervisor came up during interviews, workshops, and surveys. Survey data revealed that support from one’s immediate supervisor was important to being willing to work past 65 years of age (i.e., ordinary retirement age) [22] and that less than 20% perceived that their own managers to a high degree, or very high degree, were interested in their work or were sufficiently supportive [23]. The low interest and degree of perceived support on the part of their immediate supervisor is noticeable, considering that both the European “Framework Directive” [27] and Swedish legislation [28–30] stipulate that all public and private employers are legally obliged to protect their employees from work-related hazards that may be detrimental to health and safety.

Interestingly, many school principals expressed an ambivalent attitude, as they often desired increased regulation of their mission and more transparent role descriptions, while simultaneously requesting more autonomy. Less conspicuous, given their situation, is that they are in favour of long-term initiatives that seek to eliminate uncertainty, reduce workload, and ensure support and resources by, for example, clarifying work planning, simplifying rules and principles for documentation, improving relations to school owners and politicians, as well as facilitating access to increased support from principals, principal colleagues, local management, combined with asking for support for managing information flow, IT functions, finances, and complaints. Thus, some preventive actions target psychological aspects such as uncertainty, values, competences, and the school principals’ personal motivation and ability to prioritize and delimit work and private life, while others target the extra-psychological factors and external circumstances in each situation. Arguably, some proposals are within the school principals’ sphere of influence and control, whereas other proposals are beyond the boundaries of individual, school unit, and school owner influence. Proposals targeting the school principal as an individual partially reflect the need to learn how to prioritize between various tasks, personal needs, and the needs of others. The proposals on shared and distributed leadership were well aligned with theories of sustainable and distributed school leadership [26, 31] and recount that shared leadership is often followed by positive outcomes, such as difficult decisions becoming easier, more measured, and better for the organization, and may result in feelings of relief and of not being isolated [32].

### Methodological considerations

The fact that the school principals’ proposals for preventive actions were collected immediately prior to, or during, the Covid-19 pandemic is unlikely to have biased the results or invalidated them for future use. During the group interviews and workshops, almost no time was spent on issues related to the pandemic, and the framework for organizing education has not changed since [9].

That the qualitative data was collected with various methods such as open-ended survey responses, recordings of both group interviews and workshops as well as Mentimeter responses during other workshops, generated data of varying amount and quality. Both the open-ended survey responses and the Mentimeter responses were characterised by brief sentences, phrases, and single words whereas the group interviews and the three workshops with re-invited group interview participants gave longer and more coherent narratives. Given the present context and aim to identify proposals for suitable target areas and preventive actions that may decrease school principal’s workload and improve their mental health most data were of sufficient quality. Interestingly, single word responses in both the survey and Mentimeter data, whom as stand-alone items appeared uninformative, often acted in concert across individual participants to paint a picture of a theme or problem area that other school principals had explained with more words in the survey or in the group interviews and workshops. Nonetheless, as previously mentioned, the workshop data which was developed against the backdrop of survey and group interview data was given a greater weight in the final selection of proposals.

Despite the generally low survey response rate (presumably because of over-coverage inasmuch as job turnover, combined with the use of a compiled census that covered nine years, likely caused us to invite individuals who no longer represented the target population when invited in 2018), the school principals in the surveys, group interviews, and workshops appear to be representative of Swedish school principals as regards age, gender, and school owner [33–35], although official sources often merge school principals with other adjacent managers. Furthermore, our aggregated analysis is primarily based on the school principals’ personal attributions and statements and gives a composite picture, indicating that the individual rationalities of school principals may contradict each other. Sometimes the attributions and statements can be corroborated with other data, for example, our observations in the two surveys that role conflicts and not having managerial support are associated with signs of reduced mental health [21] and even turnover intentions [20]. At other times, however, there is no corroborating data beyond the fact that multiple school principals have independently identified and attributed the same presumed cause-and-effect relationships.

## Conclusion

The considerable number of proposals and their content reveal that preventive actions are needed at all levels of the educational system and that the school principals’ personal resources matter in improving their work situation and mental health. The results also underscore the importance of having a deep understanding of what problems to address, and in which context they unfold, before engaging in preventive actions.

## Data Availability

Consistent with the study protocol approved by the Regional Ethical Review Board, anonymized data are stored locally at Lund University, Lund, Sweden. In accordance with the ethical approval, crude data are not to be published on the internet. Access to data will be granted to eligible researchers who wish to audit our research. Requests should be directed to the corresponding author.
